# RETRACTION: Risk Factors for Postoperative Surgical Site Wound Problems after Metastatic and Primary Spine Tumour Surgery: A Meta‐Analysis

**DOI:** 10.1111/iwj.70218

**Published:** 2025-02-11

## Abstract

**RETRACTION**: ZhuJ. , SiM. , and HuangZ. , “Risk Factors for Postoperative Surgical Site Wound Problems after Metastatic and Primary Spine Tumour Surgery: A Meta‐Analysis,” International Wound Journal 20, no. 8 (2023): 3006–3014, 10.1111/iwj.14175.37118927 PMC10502245

The above article, published online on 28 April 2023, in Wiley Online Library (http://onlinelibrary.wiley.com/), has been retracted by agreement between the journal Editor in Chief, Professor Keith Harding; and John Wiley & Sons Ltd. Following an investigation by the publisher, all parties have concluded that this article was accepted solely on the basis of a compromised peer review process. In addition, the investigation found significant textual overlap between the methods and discussions sections of this article and another article by different authors [Xue et al. 2023 [https://doi.org/10.1111/iwj.14136]). The editors have therefore decided to retract the article. The authors did not respond to our notice regarding the retraction.

**RETRACTION**: J. Zhu , M. Si , and Z. Huang , “Risk Factors for Postoperative Surgical Site Wound Problems after Metastatic and Primary Spine Tumour Surgery: A Meta‐Analysis,” International Wound Journal 20, no. 8 (2023): 3006–3014, 10.1111/iwj.14175.37118927 PMC10502245

The above article, published online on 28 April 2023, in Wiley Online Library (http://onlinelibrary.wiley.com/), has been retracted by agreement between the journal Editor in Chief, Professor Keith Harding; and John Wiley & Sons Ltd. Following an investigation by the publisher, all parties have concluded that this article was accepted solely on the basis of a compromised peer review process. In addition, the investigation found significant textual overlap between the methods and discussions sections of this article and another article by different authors [Xue et al. 2023 [https://doi.org/10.1111/iwj.14136]). The editors have therefore decided to retract the article. The authors did not respond to our notice regarding the retraction.

